# Critical roles of FAM134B in ER-phagy and diseases

**DOI:** 10.1038/s41419-020-03195-1

**Published:** 2020-11-16

**Authors:** Jie Mo, Jin Chen, Bixiang Zhang

**Affiliations:** Hubei Key Laboratory of Hepato-Pancreato-Biliary Diseases; Hepatic Surgery Center, Tongji Hospital, Tongji Medical College, Huazhong University of Science and Technology; Clinical Medicine Research Center for Hepatic Surgery of Hubei Province; Key Laboratory of Organ Transplantation, Ministry of Education and Ministry of Public Health, Wuhan, Hubei 430030 P.R. China

**Keywords:** Cancer, Autophagy

## Abstract

FAM134B (also called JK-1, RETREG1), a member of the family with sequence similarity 134, was originally discovered as an oncogene in esophageal squamous cell carcinoma. However, its most famous function is that of an ER-phagy-regulating receptor. Over the decades, the powerful biological functions of FAM134B were gradually revealed. Overwhelming evidence indicates that its dysfunction is related to pathophysiological processes such as neuropathy, viral replication, inflammation, and cancer. This review describes the biological functions of FAM134B, focusing on its role in ER-phagy. In addition, we summarize the diseases in which it is involved and review the underlying mechanisms.

## Facts

- ER-phagy is a major cellular degradation system that is involved in ER fragments and ER-resident protein clearance, which plays an important role in maintaining ER homeostasis.
- FAM134B is an important ER-phagy receptor. It regulates ER-phagy and participates in some ER-phagy-related processes. It is also a *cis*-Golgi protein, but its specific role as a Golgi protein has not been elucidated.
- FAM134B plays dual roles in cancer either as an oncogene or as a tumor suppressor. It might directly regulate cancer cell apoptosis through acting as an ER-phagy receptor.

## Open questions

- Does FAM134B participate in autophagy only as an ER-phagy receptor? Is it involved in the formation of ER-derived phagophore?
- What is the functional difference between FAM134B-1 and FAM134B-2? What determines their differences in expression?
- What is the specific role of FAM134B as a Golgi matrix protein?
- Does FAM134B play a role in cancer by regulating ER-phagy?

## Introduction

FAM134B (also called JK-1, RETREG1), belonging to a family with sequence similarity 134, is the first endoplasmic reticulum (ER)-phagy (reticulophagy) receptor discovered^1^. In 2001, FAM134B was first discovered as an oncogene in esophageal squamous cell carcinoma (ESCC). Over two decades after the initial discovery, the powerful biological functions of FAM134B have been gradually revealed. The most well-known one is regulating ER-phagy^2^. The protein has two important domains—the LC3-interacting region (LIR) and the reticulon-homology domain (RHD). The LIR is responsible for binding with autophagosome while the RHD senses and induces ER membrane curvature. Based on this function, FAM134B participates in many ER-phagy-related processes such as hepatic ER-phagy, quality control of procollagens, ER-to-lysosome-associated degradation (ERLAD), reticulo-mitophagy, and preadipocyte differentiation^3–7^. Moreover, FAM134B also acts as a *cis*-Golgi protein, although its specific function in the Golgi apparatus is not quite clear^8^.

Over the last few years, dysfunction of FAM134B has been reported to be involved in many diseases, including neuropathy, viral infection, vascular disease, inflammation, and cancer^8–13^. FAM134B-induced neuropathy and viral infection are confirmed to be correlated to its function in ER-phagy regulation. In cancer biology, FAM134B shows opposite effects in different cancer types, acting either as an oncogene or a tumor suppressor^13,14^. It involves in regulation of cancer cell cycle, proliferation, apoptosis, metastasis, etc., and is correlated with PI3K/AKT and WNT/β-catenin signaling pathways.

In this review, we meticulously presented the structure of FAM134B and described how it regulates ER-phagy in detail. Then, we listed the biological processes it participated in, which are mostly based on its ER-phagy receptor functions. At the same time, we summarized FAM134B-related diseases and reviewed, to the extent possible, the mechanisms by which FAM134B mediates these diseases. Finally, we detail the role FAM134B plays in tumors, as well as important findings from our group.

## Overview of FAM134B

### Structure of FAM134B

**Fig. 1:**
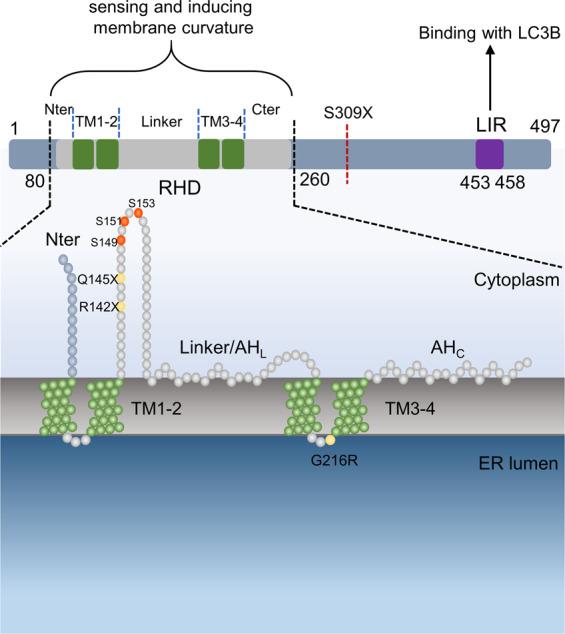
FAM134B is characterized by two important domains: the RHD and the LIR. The RHD contains two transmembrane segments (TM1–2, TM3–4, green), which are bridged by a flexible linker, and two additional terminal segments. The C-terminal segment and partial linker form amphipathic helices (AHL, AHC, gray). Three phosphorylation sites (S149, S151, and S153; orange) in the RHD are involved in protein oligomerization. Mutations of Q145 and cleavage sites of ZIKV R142X reside in the RHD, producing a truncated protein (yellow). The genetic variant G216R is a gain-of-function mutant causing excessive ER-phagy (yellow). The S309X product lacks the LIR motif so that it cannot interact with LC3-like proteins (red dotted line).

FAM134B is a 54kaD protein encoded by the *FAM134B* gene located in the chromosomal region 5p, which contains several potential oncogenes and is amplified in many tumors types^13,15,16^. The protein contains 497 amino acids with two long hydrophobic segments and a C-terminal coiled-coil domain^17^. Notably, it is characterized by the presence of two special domains: the LIR and the RHD^2,17^. The LIR motif (residues 453–458, C-terminus), which binds to autophagy modifier proteins LC3/GABARAP family, is a common domain of selective autophagy receptors^2^. Depletion or mutations of this area abolish its function in ER-phagy and result in many diseases including neuropathy and viral infections^2,9,10^. The RHD (residues 80–260, N-terminus), is also found in other ER-shaping proteins and contains four important parts. Two large transmembrane segments (TM1–TM2 and TM3–TM4) form hairpin structures that anchor the protein into the ER membrane, while two cytoplasmic amphipathic helices (AH_L_ and AH_C_) strongly interact with the cytoplasmic leaflet and flank the TM3–TM4 segment on both sides. Through the RHD, FAM134B can sense and induce membrane curvature^17^. Furthermore, the RHD contains three phosphorylation sites (S149, S151, and S153) involved in FAM134B oligomerization. Interruption of RHD disrupts the ER-phagy process and can lead to the development of disease^18^. In pigs, an m^6^A site targeted by YTHDF2 is located at 1358 bp. The loss of this site leads to abnormalities in fat deposition and adipogenic differentiation^19^ (Fig. 1).

Gene *FAM134B* produces two transcripts. Isoform 1 contains nine exons whereas isoform 2 consists of 6, encoding a 356-amino acid protein (∼39 kDa). Protein FAM134B-2 is an N-terminally truncated isoform with just one transmembrane domain. Because it lacks the RHD, it cannot control the size of ER^3^. In mice, the expression of FAM134B-1 in brain tissue was much higher than that of FAM134B-2^3^. However, FAM134B-2, and not FAM134B-1, is dramatically increased in liver and peripheral tissues upon starvation and participates in starvation-induced hepatic ER-phagy^3^.

### Cellular localization of FAM134B

To date, FAM134B is identified in the ER, Golgi, and nucleus^2,8,20^. In the ER, it anchors on ER sheets through its two transmembrane segments and acts as an ER-phagy receptor that maintains cellular homeostasis^2,17^. In general, sheets correspond to rough ER (RER) and tubules to smooth ER (SER)^21^. However, in the liver, FAM134B-2 is expressed in both RER and SER, but more abundant in SER^3^. This is related to its regulation of ER-phagy of secretory proteins such as apolipoprotein C-III in liver^3^. A series of studies demonstrate that FAM134B co-localizes with the *cis*-Golgi network and possesses typical characteristics of Golgi-resident matrix proteins^8^. Golgi matrix proteins are thought to be responsible for the complex structure of Golgi by shaping and tethering its membrane stacks. Depletion of FAM134B in DRG and N2a cells leads to alterations of Golgi structure, along with impairment of proliferation, apoptosis, and larger neuron size^8^. Interestingly, in colorectal cancer cells, FAM134B is not only located in the cytoplasm but also present in the nucleus^20^. However, its function in the nucleus is still unclear.

## Biological functions of FAM134B related to ER-phagy

### ER-phagy receptor

Autophagy is a major intracellular degradation mechanism that helps maintain the homeostasis and metabolism of eukaryotic cells both under basal conditions and a variety of stresses^22^. Canonical autophagy (macroautophagy) begins with the formation of a double-layered membrane called a phagophore. Then, the phagophore engulfs cytoplasmic materials and closes to form a vesicle called the autophagosome. The cargo in the autophagosome is transported to the lysosome and then degraded in the lysosome^23,24^. In the beginning, autophagy was considered to be a non-selective bulk degradation process that is activated during nutrient starvation. However, numerous studies showed that the process of substrate degradation can be highly selective when facing other types of stresses, such as damaged organelles or aggregated proteins^25^. This highly selective process is defined as a selective autophagy. Selectivity is largely achieved through the interaction of autophagy receptors with autophagosome-associated LC3, which links autophagy substrates to the autophagosome. The ER is fragmented and delivered to the lysosome for degradation via a specific type of autophagy^23^. We call this selective process ER-phagy. The ER is the largest membrane-bound organelle in mammalian cells, and it is involved in multiple cellular processes^26^. It also plays an important role in the quality control of newly synthesized proteins by two classical pathways: the unfolded protein response (UPR) and the ER-associated protein degradation pathway (ERAD). The former manages the expression of a vast number of genes by at least three distinct branches to maintain ER homeostasis^27^, while the latter recognizes abnormally folded polypeptides and facilitates their translocation into the cytoplasm for proteasomal degradation^28^. Of note, ER-phagy is another major cellular degradation system that is involved in ER fragments and ER-resident protein clearance. Besides, the ER also initiates bulk autophagy via forming a double-layered membrane of autophagosomes^22^. Therefore, the ER plays a dual role in autophagy, as it can be both the initiator and the target of the progress.

**Fig. 2:**
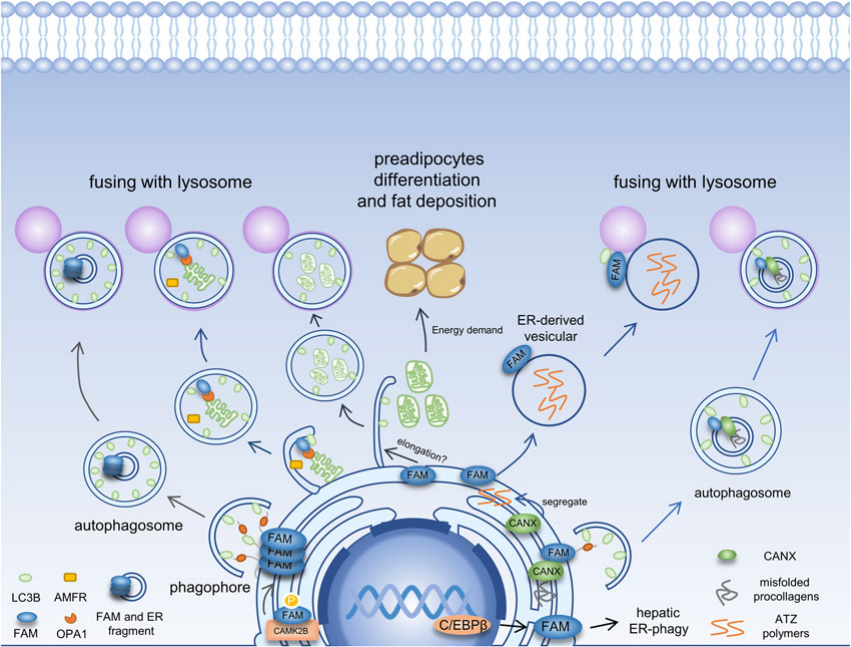
In ER-phagy, FAM134B senses and induces ER membrane curvature via the RHD. In the meantime, LIR binds to the autophagosome generating a pulling force, so that the ER membrane forms vesicles, which pinch off and are then engulfed by the phagophore. CAMK2B phosphorylates FAM134B leading to oligomerization, ER fragmentation, and ER-phagy. High levels of AMFR lead to destabilized OMM and exposed IMM. FAM134B promotes the formation of the mitophagophore by interacting with OPA1. Besides, by potentiating mitophagy, it improves preadipocytes differentiation. CANX recognizes misfolded procollagens and interacts with FAM134B. Next, FAM134B binds to the autophagosome and delivers cargos to the lysosome for degradation. ATZ polymers are degraded through the ERLAD pathway. In this process, FAM134B helps the polymers enter the vesicles from the ER domain, and promotes the docking of these ER-derived vesicles to lysosomes. CANX segregates ATZ polymers into ER subdomains and forms a CNX:FAM134B:LC3B complex for the next step. In the liver, starvation induces C/EBPβ expression, which then increases the expression of FAM134B-2. FAM134B-2 interacts with LC3B and its lysosomal degradation is increased under starvation, indicating that it plays a role in starvation-induced hepatic ER-phagy.

The process of selective autophagy is mediated by autophagy receptors, which recognize the cargo and the autophagosomal membrane through their LIR^29^. There are six known ER-phagy receptors, among which FAM134B was the first one to be identify^2,23,30–32^. Disruption of FAM134B led to a striking ER expansion and increasing in CLIMP-63-positive ER sheets. In contrast, Overexpression of FAM134B led to fragmentation and pronounced morphological changes of ER, and these FAM134B-positive fragments were co-localized with LC3B and eventually delivered to lysosomes for degradation. However, mutation or deletion of the LIR amino acid sequence abolished the interaction of FAM134B with LC3-like proteins, causing failure to induce ER-phagy^2^. It suggests that FAM134B-induced ER-phagy is mediated by the interaction between FAM134B and LC3B via the LIR motif (Fig. 2). Moreover, two major autophagy regulators, ATG5 and BECN1, are essential for ER fragmentation and degradation induced by FAM134B overexpression, suggesting that FAM134B-induced ER-phagy is dependent on the core macroautophagy machinery. However, FAM134B deficiency has little effect on the autolysosome number, turnover of long-lived proteins, as well as protein levels of autophagy marker such as LC3B and p62, suggesting that FAM134B is not required for bulk autophagy or other types of selective autophagy^2^. Thus, FAM134B may act as a general autophagy receptor for ER-phagy and contribute to ER turnover (Fig. 2).

During ER-phagy, ER membranes will form budding vesicles, which are subsequently swallowed by a phagophore. This process requires FAM134B-RHD-meditated ER membrane remodeling. The RHD is characterized by two TM segments (TM1–2 and TM3–4) and two cytoplasmic amphipathic helices (AH_L_ and AH_C_) (Fig. 1)^17^. The topological structure of RHD, which is determined by TM segments and cytoplasmic amphipathic helices, strongly curves the ER bilayer. This membrane deformation can be amplified by clusters of FAM134B-RHD. TM hairpins are crucial elements for membrane binding, and at least one TM hairpin is required for stable binding and anchoring into lipid membranes. Both amphipathic helices and TM3–TM4 play a crucial role in liposome remodeling, whereas TM1–TM2 is not required^17,33^. During ER-phagy, FAM134B-RHD first senses and induces positive curvature in the ER membrane via the TM hairpins and AHs. This mediates FAM134B-RDH clustering, magnifying the membrane deformation and producing highly curved ER membrane segments. In the meantime, FAM134B binds to LC3 on the autophagosome to create a strong pulling force. Subsequently, the ER membrane forms vesicles that pinch off, and are then engulfed by the phagophore^2,17^. Therefore, by sensing and inducing membrane curvature via RHD and directly binding to the autophagosome via the LIR, FAM134B perfectly combines two functions in the process of autophagy. Interestingly, the formation of FAM134B oligomers via RHD is required for ER membrane fragmentation and ER-phagy. Three potential phosphorylation sites (serine 149, serine 151, and serine 153) within the cytoplasmic linker that bridges two TM hairpins of the RHD are essential for FAM134B oligomerization and ER fragmentation^18,34^. Moreover, CAMK2B, a calcium/calmodulin‐dependent protein kinase, phosphorylates FAM134B at S151 to promote oligomerization, ER fragmentation, and ER-phagy. Therefore, when cells are subjected to continuous chemical stimulation, oxidative stress, or calcium overload, the ER is challenged by the accumulation of misfolded proteins which is termed as ER stress^35^. Under ER stress, activated CAMK2B induces ER oligomerization, ER fragmentation, and ER-phagy to restore ER homeostasis by the phosphorylation of FAM134B at serine 151. The latter subsequently induces ER oligomerization, ER fragmentation, and ER-phagy to restore ER homeostasis by the phosphorylation of FAM134B at serine 151. In addition, G216R within the RHD of FAM134B dramatically enhanced FAM134B oligomerization, ER scission, and ER-phagy. Excessive ER-phagy by the mutation of FAM134B^G216R^ triggers sensory neuronal cell death^18^. Microphthalmia-associated transcription factors (MiTF/TFE) promote FAM134B expression via directly binding to the CLEAR site that is located in the third intron of the FAM134B gene^36^. In chondrocytes, prolonged starvation and FGF signaling, a critical regulator of skeletogenesis, activate TFEB/TFE3–FAM134B axis and induce ER-phagy to promote skeletal development^36^. These two studies advanced our understanding of how extracellular or intracellular signals are transduced to trigger ER-phagy. Moreover, ER-phagy may also play a dual role in cell survival. When excess misfolded proteins accumulate in the ER lumen, ER stress would activate moderate ER-phagy to remove damaged ER to restore ER homeostasis. However, excessive ER-phagy induces cell death by inducing apoptosis. ATL2 (ER-integral membrane protein 2) acts downstream of FAM134B. It might contribute to separate FAM134B-marked ER via remodeling ER membrane^37^.

In summary, these works provide novel insights into the biological function of FAM134B acting as an ER-phagy receptor. Future research directions should focus on the role of FAM134B in ER stress-related diseases including diabetes, nonalcoholic fatty-liver disease, Parkinson’s, and Alzheimer’s diseases and cancers.

### Regulator of starvation-induced hepatic ER-phagy

The liver is a central metabolic organ that is closely related to nutrition. It has been confirmed that nutrient starvation triggers ER-phagy in liver cells regulated by transcription factors such as CREB, PPARs, FXR, and C/EBP^38–40^. In mouse hepatocytes, the expression of a small-size FAM134B (FAM134B-2) was found to be increased upon starvation. FAM134B-2 is an N-terminally truncated isoform of FAM134B that retains the LIR motif but lacks the RHD. However, mouse hepatocytes do not express FAM134B-1, and this may not be a post-translational truncation but rather stem from another isoform of FAM134B mRNA produced by alternative transcription. There is evidence that C/EBPβ (LAP) mediates starvation-induced FAM134B-2 expression. Starvation induces C/EBPβ expression and increases its recruitment to the FAM134B-2 promoter. In the ER, FAM134B-2 interacts with LC3B, and its lysosomal degradation is increased under starvation, indicating that it acts as a hepatic ER-phagy receptor. In this process, FAM134B-2 regulates the ER-phagy of secretory proteins such as apolipoprotein C-III (ApoCIII) but does not affect bulk ER turnover^3^ (Fig. 2). This work shed light on novel insights into the role of FAM134B-2 in starvation-induced selective ER-resident protein degradation in the liver. We suppose that FAM134B-2 may play a different role in ER-stress-related diseases due to lacking RHD domain.

### Quality control of procollagens

To maintain ER homeostasis, most misfolded proteins in the ER lumen are transported into the cytoplasm for degradation by the proteasome, a process called ERAD^41^. However, not all the misfolded proteins are cleared via ERAD. Procollagen is the most abundant protein in animals which are synthesized in the ER and degraded by ER-phagy if they are misfolded^30,31^. In this process, the ER-resident lectin chaperone Calnexin (CANX, CNX) recognizes misfolded fractions and interacts with FAM134B, which then binds to autophagosome and delivers misfolded procollagens along with CANX to the lysosome for degradation. The interaction between CANX and FAM134B is stable and not modulated by procollagens, suggesting that the interaction between procollagens and CANX might contribute to RHD conformation change that increases ER membrane curvature, promoting vesicle formation (Fig. 2). In Fam134b^−/−^ MEFs, both Col1a1 and Col1a2 peptide chains are significantly increased, suggesting that procollagens are the main clients of FAM134B-mediated ER-phagy^4^. The quality control of procollagens implies that FAM134B plays a role at the juncture of ER protein quality control and selective autophagy of specific proteins.

### A member of ERLAD

Some misfolded proteins like the disease-causing polymerogenic E342K (ATZ) variant of alpha1 antitrypsin (AT), various serpin mutants, and mutant hormone receptors, are proteasome-resistant misfolded proteins that must be degraded via the lysosomal system as well^42–44^. However, unlike procollagen, they are not eliminated by the autophagy pathway either^43,45^. Thus, there may be another protein clearance pathway for those “autophagy-proof” proteasome-resistant proteins By tracing ATZ polymers, a new pathway called the ERLAD pathway has been discovered^5^. ERLAD does not rely on the UPR or autophagy and uses single-membrane, ER-derived vesicles to transport the polymers to lysosomes by membrane fusion. FAM134B takes part in this mechanism by helping polymers enter the vesicles from the ER domain and promoting the docking of ER-derived vesicles to lysosomes. Notably, CANX also plays a crucial role in this process by segregating ATZ polymers into ER subdomains and then forming a CANX:FAM134B:LC3B complex for the next step^5^ (Fig. 2). This is the second interaction between CANX and FAM134B, although their functions both changed. FAM134B plays a role as receptors in both ERLAD and ER-phagy. However, in ERLAD, FAM134B mediates the association of ER-derived vesicles and RAB7/LAMP1-positive endolysosomes by binding membrane-bound LC3B. In ER-phagy, FAM134B binds to membrane-bound LC3B to promote the ER-derived vesicles docking to autophagosomes.

### Participating in reticulo-mitophagy—a dual organellar turnover mechanism

ER E3 ligase AMFR targets outer mitochondrial membrane (OMM) proteins and MFNs (mitofusins) for ubiquitination and degradation^46^. Under some circumstances like the increase of cytosolic Ca^+^, the level of AMFR is elevated^46^. High levels of AMFR increase degradation of MFNs and OMM, thus exposes the inner mitochondrial membrane (IMM), triggers another common model of selective autophagy–mitophagy^46^. The destruction of OMM brings the IMM close to the ER^7^. FAM134B located in the ER membrane interacts with one of the IMM proteins, OPA1, pulls the ER forming mitophagophore to wrap mitochondria^7^. In this process, AMFR along with the destroyed mitochondria are delivered to the lysosome for degradation^7^ (Fig. 2). Therefore, reticulo-mitophagy cleared destroyed mitophagy and simultaneously eliminated excessive AMFR, eventually reducing mitophagy to reestablish homeostasis in cells. This provides new insight into the role of FAM134B in cellular stress and autophagy. Under cellular stress, the ER and mitochondria increase their communications with each other, and FAM134B is the bridge between them. The above three examples demonstrate that FAM134B not only plays a role in ER-phagy, but also participates in a variety of pathways to maintain cell homeostasis.

### Promoting preadipocyte differentiation and adipogenesis

Autophagy plays an important role in the differentiation of many cells, including erythrocytes, lymphocytes, and adipocytes^47–49^. Mitophagy promotes the development of red blood cells and might contribute to adipocyte differentiation^50^. Mitochondria are needed to meet the dramatically increased energy needs in the early stage of adipocyte differentiation and are then rapidly cleared to develop the mature cell structure^6,51,52^. This clearance is possibly accomplished by mitophagy. As an ER-phagy receptor, FAM134B improves preadipocyte differentiation by potentiating mitophagy^6^. Its role in this process might be to provide material from the ER to facilitate the elongation of the autophagosome. Furthermore, FAM134B plays a functional role in a fat deposition by increasing the mRNA levels of proteins involved in adipogenesis and decreasing the levels of lipolytic enzymes^53^. Moreover, an m^6^A site of FAM134B is targeted by YTHDF2, which leads to mRNA degradation and decreased protein levels. The loss of this m^6^A site enhances the ability of FAM134B to promote adipogenesis and preadipocyte differentiation^19^ (Fig. 2).

## FAM134B and nonneoplastic diseases

### Virus replication

**Fig. 3:**
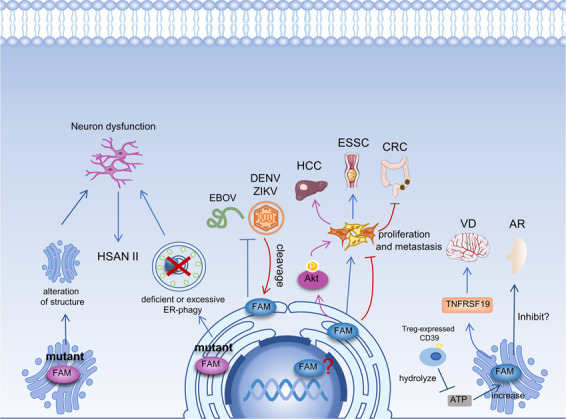
Mutations of FAM134B disturb ER-phagy and may cause alterations of Golgi structure. Related pathological processes might break the homeostasis of neural cells, leading to the progression of HSAN II. FAM134B obstructs the replication of DENV, ZIKV, and EBOV. Consequently, DENV and ZIKV cleave FAM134B to subvert ER-phagy. FAM134B acts as an oncogene in HCC and ESCC, but it plays a tumor suppressor role in CRC. In HCC, FAM134B activates the Akt signaling pathway, promoting cell proliferation and metastasis. In CRC, FAM134B is not only located in the cytoplasm but also in the nucleus. However, the role it plays in the nucleus is still unclear. FAM134B and TNFRSF19 show a strong synergy in vascular dementia. Their effect may be related to their roles in the Golgi apparatus. In AR, the expression of CD39 on Treg cells is negatively correlated with the expression of FAM134B on monocytes. The CD39 expressed by Treg cells can hydrolyze extracellular ATP to suppress asthmatic airway inflammation. However, ATP can increase the expression levels of FAM134B in CD4^+^ blood monocytes. Thus, ATP may be a link between CD39 and FAM134B in AR.

The ER is a large membrane-bound organelle that is involved in protein synthesis and quality control as well as lipid synthesis^26^. Because of these characteristics, the ER is utilized by some viruses during their life cycles. For example, flaviviruses not only replicate their positive-strand RNA genomes in an invagination made of the ER membrane but also assemble their virions in the ER and then secrete from it for maturation^54,55^. To maintain homeostasis, host cells will activate a series of processes to protect the ER, including ER-phagy. FAM134B has been reported to be involved in the infection process of the Ebola virus (EBOV) and flaviviruses, serving as an ER-phagy receptor^9,10^ (Fig. 3).

Research on EBOV showed that its replication increased in the absence of FAM134B along with higher levels of VP40 and GP, demonstrating that FAM134B depletion contributes to EBOV replication. Moreover, in FAM134B^−/−^ cells, there was an increased accumulation of nucleocapsids in cytosolic inclusion bodies compared to wild-type cells. All these findings indicate that FAM134B impedes the replication of EBOV^9^ (Fig. 3). Dengue (DENV) and Zika (ZIKV) are mosquito-borne flaviviruses that severely threaten global public health. Flavivirus infection promoted ER expansion by DENV NS3-induced relocalization of fatty acid synthase and de novo lipid synthesis at sites of viral replication. The process contributes to the formation of replication of vesicles. However, FAM134B-mediated ER-phagy restricts virus-induced ER expansion and eliminates ER-associated viral proteins, eventually leading to a limitation of viral replication. Depletion of FAM134B results in a 7-fold increase of both DENV and ZIKV virus-RNA. Interestingly, DENV and ZIKV can manipulate the process of ER-phagy by cleaving FAM134B to promote their replication. It is found that the cleavage takes place in the cytoplasmic part of RHD by the virally encoded protease complex NS2B3, which produces 2 fragments (N- and C-terminal). The C-terminal fragment retains the LIR and the ability to interact with LC3B but is unable to form oligomers. Thus, membrane curvature and subsequent budding of ER-derived vesicles are inhibited. Additionally, the C-terminal fragment exhibits decreased localization to autophagosomes. Collectively, virally encoded proteases NS2B3-mediated cleavage of FAM134B can subvert ER-phagy to facilitate viral replication^10^ (Fig. 3).

Autophagy can be an ally or an enemy to viruses depending on the virus itself and the cellular state^56^. Accumulations of the EBOV GP in the ER induce cytotoxicity by disrupting cellular homeostasis^57^. To restore cell homeostasis, host cells may initiate autophagy to accelerate the turnover of cellular contents and organelles required for viral replication and assembly, which is detrimental to viruses. Thus, autophagy seems to have an adverse effect on multiple facets of the replication of these two viruses. Moreover, FAM134B dysfunction can provide more ER and RER for viral protein synthesis, or even subvert ER-phagy. Although the link between autophagy and viruses has not been elaborated yet, linking the role of FAM134B dependent ER-phagy and virus replication may provide fruitful new targets for antiviral therapy. Since FAM134B acts as a barrier for viral replication, overexpressing it or preventing it from virus-RNAi mediated silencing and cleavage might be fruitful strategies for antiviral interventions.

### Neuropathy

Hereditary sensory and autonomic neuropathies (HSANs) are a heterogeneous group of disorders that affect peripheral sensory and autonomic neurons^58^. HSAN II is a rare autosomal recessive disease that is characterized by prominent distal sensory loss and mutilations, acropathy, and disease onset in the early decades of life^58^. There are many case reports and studies which confirmed that mutations of FAM134B cause severe HSAN II.

A report of a Saudi Arabian patient from an index family with HSAN II firstly found a loss-of-function mutation of FAM134B, (c.926 C > G; p.S309X). This mutation may cause nonsense-mediated decay (NMD) and eventually lead to a nonfunctional gene product^8^. Subsequently, another analysis of 75 unrelated individuals diagnosed with HSAN showed different mutations in FAM134B, including a 2-bp deletion causing a frameshift (p.P7GfsX133), a nonsense mutation (p.Q145X), and a mutation in the splice-donor consensus site of intron 7 (c.873 + 2 T > C)^8^. Afterward, many case reports show the same or different FAM134B mutations in patients with HSAN II^59–63^. For a more intuitive overview, we summarize some mutations of FAM134B in HSAN II in Table 1.

**Table 1:** Mutations of FAM134B in HSAN II.

Research group	Origin	Mutation	Amino acids changes	Mutation type	Characteristic
Kurth et al.^7^	Saudi Arabia	c.926 C > G	p.S309X	Nonsense	Lacks LIR remains RHD
		c.17delCT	p.P7GfsX133	Deletion	frameshift
		c.433 C > T	p.Q145X	Nonsense	Lacks LIR and partial RHD
		c.873 + 2 T > C	-	Deletion	Mutant in splice-donor consensus site
Davidson et al.^61^	United Kingdom	c.433 C > T	p.Q145X	Nonsense	Lacks LIR and partial RHD
		c.646 G > A	p.G216R	Substitution	Residing in RHD, gain-of-function mutant
Murphy et al.^62^	Somalia	c.471 C > T	p.Q145X	Nonsense	Lacks LIR and partial RHD
Ilgaz Aydinlar et al.^63^	Turkey	c.826delA	p.S276VfsX8	Deletion	Frameshift
Wakil et al.^64^	Saudi Arabia	c.926 C > G	p.S309X	Nonsense	Lacks LIR remains RHD
Falcao de Campos et al.^65^	Turkey	c.896_897delAA	p.L299RfsX6	Deletion	Frameshift
	Portugal	c.1426del	p.G476RfsX57	Deletion	Frameshift

In aged FAM134B^−/−^ mice, some symptoms associated with loss of small nociceptive and myelinated axons appeared. Additionally, their sensory axon numbers were significantly reduced. Ultrastructural analyses revealed that ER as well as Golgi cisternae expanded in FAM134B^−/−^ mice at 10 months of age, and these changes were absent in wild-type littermates. These data suggested a role of FAM134B in sensory axon maintenance^2^.

Autophagy plays a crucial role in quality control of neurocytes and homeostasis of various postmitotic cells, especially in neurons^64^. In mice, suppression of basal autophagy in central nervous system cells causes neurodegenerative diseases, including locomotor ataxia, abnormal limb-clasping, and reduction in coordinated movement^65,66^. Furthermore, the accumulation of autophagosomes is also associated with Alzheimer’s disease^67^. This evidence suggests that autophagy has a strong connection with neurological disorders. The S309X protein lacks the coiled-coil domain and the LIR motif so that it cannot interact with LC3-like proteins, but it can still shape the ER membrane^2^. By contrast, the Q145X product lacks the LIR motif and a part of the RHD, making it neither interact with LC3-like proteins nor involved in ER membrane remodeling^2^. The G216R mutation in the RHD produces a gain-of-function mutant causing hyperactive oligomerization, aberrant ER scission, excessive ER-phagy and eventually leads to apoptotic neuronal death^18^. For FAM134B, loss of function or hyperactivity as an ER-phagy receptor may eventually lead to HSAN II. However, it may also maintain neurocyte stability through its role as a *cis*-Golgi protein (Fig. 3).

### Vascular dementia and allergic rhinitis

Vascular dementia (VD) is induced by cerebrovascular pathological changes^68^. Previous studies on this disease focused on the effects of individual genes and ignored potential epistatic effects. Kong et al. used a multifactor dimensionality reduction analysis to study 207 VD patients and 207 matched controls. The analysis results indicated that FAM134B and TNFRSF19 showed a strong synergy, even stronger than angiotensinogen and transforming growth factor-β1, which were previously reported to be related to VD^11,69^. However, there are no reports on the direct or indirect interaction of these two genes. Notably, a binding factor of TNFRSF19, tumor necrosis factor receptor-associated factor 2, was found to activate NF-κB in activated B cells through the tumor necrosis factor receptor-associated factor 1 complex fused with Golgi vesicles^70^. In addition, the fusion of TNF receptosomes with *trans*-Golgi vesicles results in the activation of acid sphingomyelinase and cathepsin D, which lead to vascular diseases^70,71^. Since FAM134B is a Golgi protein, it may participate in the fusion of TNF family proteins with the Golgi or subsequent events (Fig. 3). Furthermore, previous studies showed some common features of the two genes, with both mRNAs being regulated by vitamins, and both were found to be related to arteriosclerosis, aging, and obesity, which are strong risk factors of VD^11^.

Allergic rhinitis (AR) is an airway disease, the process of which involves complex interactions of inflammatory cells, such as CD4^+^ T cells, IgE-producing B cells, and mastocytes^72^. In this complex process, CD4^+^ Foxp3^+^ regulatory T cells (Treg) act as regulatory cells through the expression of the ectoenzyme CD39 on their surface^73^. The expression of CD39 on Treg cells is regulated by its upstream promoter SNP rs7071836. Although CD39 is also expressed on the surface of other cells, this regulation is restricted to Treg cells^12^. After assessing a Singaporean Chinese cohort, SNP rs7071836 was found to affect AR risk by epistatic interactions with SNP rs257174, which is located upstream of FAM134B and alters its expression levels in monocytes^12^. This epistatic interaction suggested an inverse relationship between the expression of CD39 and FAM134B, which was confirmed by whole blood measurements in three Caucasian cohorts of healthy individuals. However, without rs7071836, FAM134B could not affect the risk of AR. The epitasis of these two genes only drives the clinical symptoms but is not associated with atopy^12^. Extracellular ATP plays a significant role in triggering and maintaining allergic reactions by activating dendritic cells^74^. The CD39 expressed by Treg cells can hydrolyze extracellular ATP to suppress asthmatic airway inflammation^74^. Notably, ATP can increase the expression levels of FAM134B in CD4^+^ blood monocytes^12^. Thus, ATP may link CD39 to FAM134B influences AR. This study primarily indicated that FAM134B is a potential effector protein in the inflammatory response, although the precise mechanism remains unclear. It was hypothesized that FAM134B influences cytokine secretion by monocytes in response to external stimuli, such as ATP, through its role as a Golgi protein (Fig. 3).

## FAM134B and cancer

**Fig. 4:**
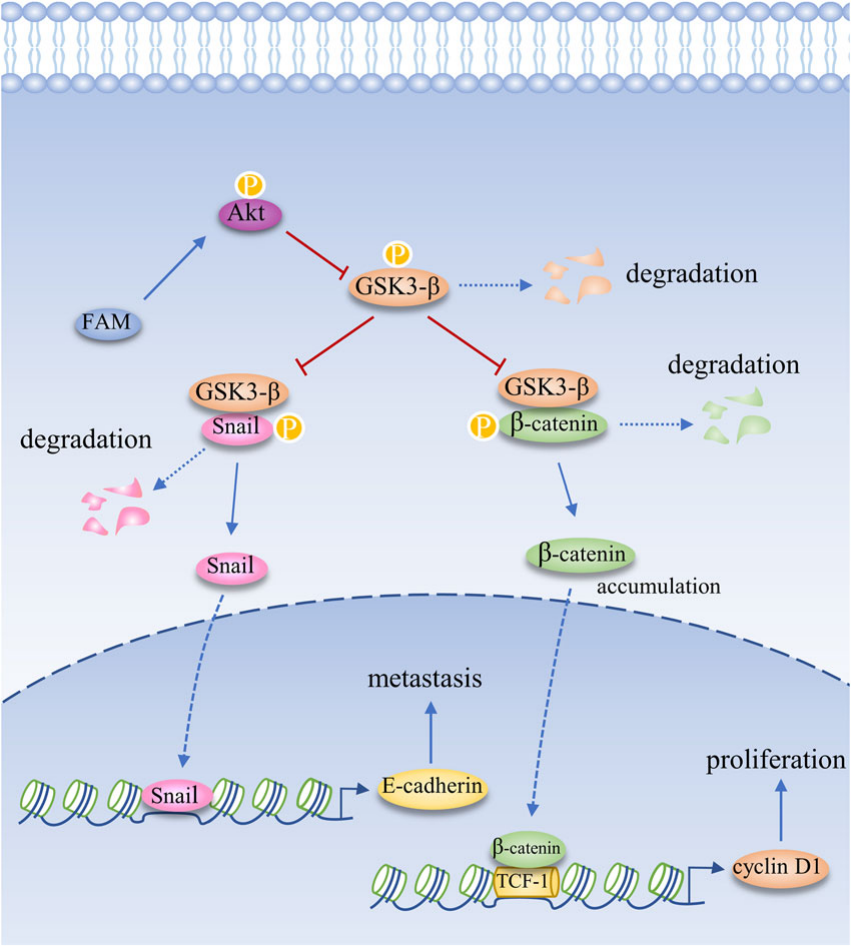
In HCC, FAM134B activates Akt to induce GSK3-β phosphorylation and subsequent dysfunction. GSK3-β phosphorylates β-catenin and Snail, leading to their degradation. Thus, by activating Akt, FAM134B inactivates GSK3-β, stabilizes β-catenin, and Snail. On the one hand, the increasing β-catenin translocates into the nucleus and interacts with TCF/LEF to increase the expression of CyclinD1 to promote cell proliferation. On the other hand, Snail suppresses E-cadherin expression via directly impinging on its promoter to trigger EMT in the nucleus.

FAM134B was first discovered as an oncogene in ESCC^1^. Over the years, studies uncovered its roles in many malignancies, including ESCC, colorectal carcinoma (CRC), breast carcinoma, and hepatocellular carcinoma (HCC)^13,20,75,76^. Based on the available results, it can be inferred that FAM134B is an oncogene in ESCC and HCC, while it acts as a tumor suppressor in colon cancer and breast cancer (Fig. 4). The mechanism by which it plays a specific role in cancer has not been thoroughly studied. Several lines of evidence indicate that it may regulate cancer cell death and apoptosis through its role as an ER-phagy receptor (Fig. 3).

FAM134B in ESCC FAM134B promotes cell proliferation and metastasis in ESCC^13,77^. Paradoxically, it showed a tendency of low expression in ESCC^13,77^. The low expression of FAM134B protein may be caused by chromosomal deletion or promoter hypermethylation. Moreover, these studies both showed that patients with high expression of FAM134B tended to be in the early stage and with high histological grade of ESCC. These seemingly contradictory correlations may have to do with the biological nature of ESCC itself. In addition, 37 mutations of FAM134B are detected in exons 4, 5, 7, 9, and introns 2, 4, 5, 6, 7, 8, most of which were homozygous and frequently present in exon 9. Notably, the two mutations c. 1137delT (alters and can truncate the protein) and p.Glu163Lys (causes an amino acid change), are likely to have increased functional significance. Mutations are more often detected in metastatic ESCC cells of lymph nodes than in primary ESCC tissues, indicating that these mutations might be involved in the progression of lymph node metastasis and a potential target for predicting lymph node metastasis^78^.

### FAM134B in CRC

Two large-cohort studies of CRC showed that the expression of FAM134B mRNA and protein was significantly lower in adenocarcinoma tissues compared to nontumor tissues. Furthermore, patients with low expression of FAM134B were more likely to have an advanced TNM stage and had a poor survival rate^79,80^. Notably, it was found that colorectal adenoma has more amplification of FAM134B than deletions compared to nontumor tissues^79,80^. The high expression of FAM134B in premalignant tumors may indicate its function in protecting cells from transformation into the malignant stage. However, mucinous adenocarcinoma, which is more aggressive and has a poorer prognosis, was found to have fewer deletions of FAM134B compared to conventional adenocarcinoma^79^. This suggests that FAM134B may be involved in other pathways of mucinous adenocarcinoma progression. Another large-cohort study of FAM134B methylation in colon tissue found that hypermethylation of FAM134B promoter was more common in CRC tissues than in adjacent nonneoplastic and adenoma tissues. Furthermore, the methylation had certain relevance for the depletion of FAM134B. Moreover, FAM134B promoter methylation was correlated with advanced TNM stages, higher pathological stages, and a poor survival rate^81^. These findings are in line with the two large-cohort studies of FAM134B expression mentioned above. Thus, we can hypothesize that promoter hypermethylation of FAM134B might be the direct cause of its downregulation in the majority of colorectal adenocarcinomas. Moreover, methylation of FAM134B is associated with perineural infiltration and lymphovascular invasion, suggesting that methylation-mediated silencing of FAM134B may promote distant metastasis^81^.

Totally, 42 nucleotide variants are observed in CRC, most of which are heterozygous and located in exons 4, 5, 7, or 9. Amongst the 42 mutations, 6 variants (c.744 C > T, c.1164 A > G, c.1112 T > C, c.1149 A > G, c.1153 G > C, and c.1107 G > C) are previously reported in ESCC, and 8 frameshift mutations may cause mRNA NMD, producing a strongly truncated protein. Mutations in exon 4 are connected with the sex of the patients and the T stage of the disease, while mutations in exon 7 are related to the presence of distant metastasis. Additionally, frequent FAM134B mutations are observed in patients with cancers characterized by microsatellite instability, implying that FAM134B may help cells maintain genetic integrity and repair DNA damage. Notably, BRAF mutations reduced FAM134B expression in CRC, suggesting that FAM134B has downstream interactions with BRAF-associated cancer pathways^82^.

In CRC, the downregulation of FAM134B leads to an increase of the growth rate, altered cell cycle, and reduced apoptosis^20,83^. The specific cell cycle change leads to the accumulation of cells in the S phase, indicating that FAM134B suppresses the synthesis phase of the cell cycle^20^. Moreover, it suppresses the metastasis of CRC via an unknown mechanism^14,20^. Importantly, FAM134B modulates the Wnt/β-catenin signaling pathway by directly interacting with EB1^83^. EB1 (MAPRE1), is a regulator of microtubule stability. It interacts with APC and aurora B kinase to regulate microtubule dynamics, cell polarity, and chromosomal instability^84,85^. Notably, APC is a tumor suppressor promoting the destabilization of β-catenin and plays an important role in the transformation of normal mucosa into adenoma^86^. The depletion of FAM134B induces the upregulation of EB1, followed by APC reduction and increase of β-catenin (both cytosolic and nuclear)^83^. Therefore, silencing or abrogation of FAM134B in colon cancer may lead to APC reduction in early stages, thus contributing to the adenoma-carcinoma transformation.

### FAM134B in breast carcinoma

Analysis of gene expression data of various gene expression synthesis (GEO) datasets for breast cancer using genetic algorithms and bioinformatics tools showed that FAM134B combined with KIF2C, ALCAM, and KIF2A may identify certain subtypes of breast cancer. In addition, higher expression of FAM134B was associated with a higher survival rate of breast cancer patients, indicating that FAM134B acts as a tumor suppressor in this type of cancer^75^.

### FAM134B in HCC

Our group firstly determined the expression of FAM134B in HCC. By studying 50 pairs of HCC tissues and nontumor tissues, we found that FAM134B was upregulated in 62% (31/50) of the HCC tissue samples. IHC microarray analysis of 122 pairs of primary HCC tissues and adjacent normal tissues showed that overexpression of FAM134B was present in 56/122 (45.9%) of HCC tumor tissues, while only 9% (11/122) showed low expression of FAM134B^76^. Additionally, overexpression of FAM134B in HCC was significantly associated with tumor size, vascular invasion, differentiation grade, cancer recurrence, portal vein tumor thrombus, shorter overall survival, and shorter disease-free survival^76^.

In HCC, FAM134B promotes proliferation and metastasis via activating Akt signaling pathway^76^. The activated Akt induces GSK3-β phosphorylation at Ser9 and subsequent inactivated GSK3-β^76^. GSK3-β is thought to phosphorylate β-catenin and snail, which targets the two proteins for ubiquitination and subsequent degradation^87,88^. Therefore, FAM134B inhibits GSK3-β activity to stabilize and accumulate the β-catenin and Snail in the cytosol by inducing AKT phosphorylation. On the one hand, the increasing β-catenin translocates into the nucleus and interacts with TCF/LEF to increase the expression of CyclinD1 to promote cell proliferation^76^. On the other hand, Snail suppresses E-cadherin expression via directly impinging on its promoter to trigger EMT in the nucleus^89^ (Fig. 4). However, the mechanism by which FAM134B activates AKT signaling needs to be further investigated.

### FAM134B and apoptosis

Apoptosis (type I cell death) is a form of programmed cell death that occurs in response to cell damage or stress as well as during normal development^90^. It can proceed via the mitochondrial pathway (intrinsic) and the death receptor pathway (extrinsic)^90^. Under stressed conditions, depletion of FAM134B leads to damaged ER and misfolded proteins accumulation in ER by dysfunction of ER-phagy, which eventually induces apoptosis and sensitizes cells to apoptosis^2,8^. In lung cancer cell line A549, decreased FAM134B accelerates the accumulation of both PARP and CASPASE 9 during starvation, whereas the level of CASPASE 8 is not changed^2^, indicating that FAM134B is a putative antiapoptotic protein influencing the mitochondrial pathway. Autophagy and apoptosis are two self-destructive processes that eliminate damaged cells or organelles^34,91^. Their relationship is complicated. It is previously reported that the lack of nutrients in cancer cell line Hela and HCT116 induces autophagy for cell survival. However, inhibition of autophagy in this scenario results in cell apoptosis^35^. Therefore, depletion of FAM134B might induce apoptosis as a consequence of ER-phagy inhibition. Conversely, decreased FAM134B levels reduced apoptosis in colon cancer^20,83^. Thus, FAM134B-induced apoptosis might be tissue-specific in different cancers.

Under other physiological milieus, autophagy can also induce a kind of cell death called autophagic cell death (type II cell death). In normal conditions, ER stress or UPR are the factors triggering autophagy. However, a small molecule Z36-induced ER-phagy results in ER stress, UPR, and cell death^92^. In this process, FAM134B along with LC3 and ATG9 is upregulated by Z36 and mediates excessive ER-phagy, leading to accelerated ER degradation and impaired ER homeostasis^92^. Moreover, overexpression of FAM134B alone in Hela is enough for inducing ER stress and cell death^92^. Collectively, FAM134B participates in both type I and type II cell death processes. The effect of FAM134B on cell death might be determined by the net balance of these two opposing pathways under different circumstances and might be cancer type-dependent.

## Conclusions

FAM134B is a powerful molecule with several intriguing functions. As an ER-phagy receptor, dysfunction of FAM134B can induce many diseases, including HSAN II and viral replication, which are strongly correlated with ER stabilization and well-balanced autophagy. In addition, VD and AR might be related to its function as a *cis*-Golgi protein. It is worth noting that the functions of FAM134B do not work independently. For instance, the occurrence of HSAN II might be a result of a combination of the dysfunction of both ER-phagy and the Golgi complex. Many studies have shown that FAM134B plays a dual role and it is a powerful molecular determinant in cancer. Unfortunately, the specific mechanisms have not been thoroughly studied to date. In HCC, we confirmed its role as an oncogene, which is contradictory to the findings in colon cancer. By activating the Akt signaling pathway, FAM134B promotes the proliferation and metastasis of HCC. With its powerful functions and important roles in the progression of diseases, FAM134B might be a potential biomarker for disease screening and diagnosis or even a target for successful new therapies.

## Data Availability

The data that support the conclusion of this review have been included in the article.
